# Prevalence, spectrum and aetiology of valvular heart disease based on community echocardiographic screening transition from different altitudes in Yunnan, China

**DOI:** 10.1136/heartjnl-2024-325221

**Published:** 2025-03-03

**Authors:** Linhong Pang, Yu Xia, Mingjing Tang, Min Ma, Hong Ran, Ziwen Zhao, Tianyu Wang, Juan Yang, Jin Li, Yunfei Zhou, Lin Duo, Zhiling Luo, Da Zhu

**Affiliations:** 1 Department of Structure Heart Center, Fuwai Yunnan Hospital, Chinese Academy of Medical Sciences, Affiliated Cardiovascular Hospital of Kunming Medical University, Kunming, Yunnan, China; 2 School of Health Policy and Management, Chinese Academy of Medical Sciences and Peking Union Medical College, Beijing, China; 3 Department of Scientific Research and Chronic Disease Management, Fuwai Yunnan Hospital, Chinese Academy of Medical Sciences, Affiliated Cardiovascular Hospital of Kunming Medical University, Kunming, Yunnan, China; 4 School of Public Health, Kunming Medical University, Kunming, Yunnan, China; 5 Department of Echocardiogram, Fuwai Yunnan Hospital, Chinese Academy of Medical Sciences, Affiliated Cardiovascular Hospital of Kunming Medical University, Kunming, Yunnan, China; 6 Department of Cardiology, Fuwai Yunnan Hospital, Chinese Academy of Medical Sciences, Affiliated Cardiovascular Hospital of Kunming Medical University, Kunming, Yunnan, China

**Keywords:** Valvular Heart Disease, Epidemiology, Cardiovascular Diseases, Echocardiography

## Abstract

**ABSTRACT:**

**Background:**

The burden of valvular heart disease (VHD) is rising rapidly globally, accompanied by substantial geographical disparities. Although altitude may influence cardiovascular system, no community-based studies have yet explored altitudinal differences in VHD epidemiology.

**Objective:**

This study aims to investigate the prevalence, spectrum and aetiology of VHD in different altitude areas.

**Methods:**

We conducted two sequential community-based echocardiography screening programmes in Yunnan Province of China and included 5059 eligible participants aged 35 years and older. The multivariable Poisson regression models with robust variance were performed to assess the association of different altitude groups with VHD and its subtypes.

**Results:**

The prevalence of overall VHD, clinically significant VHD and clinically significant regurgitant VHD was 36.7%, 2.5% and 2.4%, respectively. After stratification by altitude, the prevalence of any VHD among participants in the <2000 m, 2000–2499 m, 2500–2999 m and ≥3000 m groups was 30.4%, 40.9%, 35.0% and 44.3%, respectively. The fully adjusted models showed that the prevalence ratios for VHD in the 2000–2499 m, 2500–2999 m and ≥3000 m groups were 1.28 (95% CI 1.15 to 1.42), 1.20 (95% CI 1.02 to 1.41) and 1.34 (95% CI 1.04 to 1.72), compared with the <2000 m group. Clinically significant VHD in the <3000 m altitude group was predominantly degenerative in aetiology, whereas functionality was most prevalent in the ≥3000 m altitude group. Subgroup analysis identified some high-risk populations, including male, minority ethnicity, 60 years and older and high systolic blood pressure.

**Conclusions:**

Adults living at high altitudes have a higher prevalence risk of VHD; significant altitudinal differences exist in the characteristics and aetiology of VHD. The findings could provide insights into primary prevention and early screening for VHD in low- and middle-income countries where a majority of the population lives at high altitudes.

**WHAT IS ALREADY KNOWN ON THIS TOPIC**

- Valvular heart disease (VHD) is a rapidly growing cause of cardiovascular morbidity and mortality worldwide, with a diverse geographical distribution and a significant burden in developing countries.
- There are no community-based studies to reveal the epidemiological characteristics of VHD at different altitudes.

**WHAT THIS STUDY ADDS**

- In the two cross-sectional studies based on community echocardiography screening, we found a significantly higher risk of VHD in adults living at high altitude areas, especially at ≥2500 m.
- We also identified patients with clinically significant VHD living at <3000 m altitude attributed primarily to degenerative, whereas functional VHD was most prevalent in those residing at ≥3000 m altitude.
- Subgroup analyses showed a higher risk of VHD in males, people aged 60 years and older, ethnic minorities and people with high systolic blood pressure.

**HOW THIS STUDY MIGHT AFFECT RESEARCH, PRACTICE OR POLICY**

- Given that the majority of populations living in low- and middle-income countries reside at higher altitudes, the results of this study could provide perspective insights into early screening for VHD and improving awareness of VHD among healthcare practitioners and public education in these regions with limited healthcare resources.

## Introduction

 Valvular heart disease (VHD) is a rapidly growing leading cause of cardiovascular morbidity and mortality, and loss of physical function and quality of life worldwide,^1^ especially in low- and middle-income countries.^2^ The epidemiology of VHD varies considerably around the world. The incidence of degenerative VHD is increasing in industrialised countries; however, rheumatic VHD remains the most common worldwide^3^ and has affected approximately 41 million people.^4^ Clarifying geographical and temporal trends and changes in the epidemiology of VHD is crucial for progress in clinical practice and the development of effective primary and secondary preventive health policies.^1^

Nkomo *et al* reported VHD prevalence (2.5%) among the US 2000 population in 2006.^5^ A community-based echocardiographic screening revealed the most common VHD to be mitral regurgitation (MR, 22%) and aortic regurgitation (AR, 15%).^6^ A nationwide study from China revealed that the prevalence of moderate or greater VHD was 3.8%, with AR being the most common (1.2%), followed by MR.^7^ VHD has increasingly become recognised as a manifestation of degenerative processes related to ageing.^2^ Several existing studies have shown an increasing incidence of VHD with age, mainly due to degenerative pathophysiology.^5 6^ Globally, it is estimated that 81.6 million people live at ≥2500 m altitude.^8^ Hypoxic conditions,^9^ low temperature^10^ and large span daily temperature variability may affect the cardiovascular system to a certain degree.^11^ However, some studies also indicated that acclimatisation diminishes the risk, and hypoxia conditioning can even benefit and protect the cardiovascular system.^12^ In contrast, several population-based epidemiological studies have indicated that living at high altitudes can lead to pulmonary hypertension, stroke and left ventricular (LV) diastolic dysfunction.^13^ The role of altitude on cardiovascular system remains conflicting.^13^ There are no epidemiological studies on community-based VHD at different altitudes.

Yunnan Province belongs to the mountainous plateau terrain with an altitude range from the lowest 76.4 m to the highest 6740 m, among which the middle altitude area of 1000–3500 m accounts for 87.21% of this province’s land area.^14^ The average altitude of the province is approximately 2000 m. There is a pressing need to better understand the characteristics and burden of VHD in the different altitudes at these ideal sites. Accordingly, we conducted two community-based, high-quality, transthoracic echocardiography screening programmes at different altitudes in Yunnan Province and sought to identify altitudinal differences in the prevalence, characteristics, spectrum and aetiology of VHD in this representative population.

## Methods

### Study design and participants

The cross-sectional survey was conducted in Yunnan Province, a relatively low-income province in southwestern China (online supplemental figure S1). Data were obtained from two sequential community-based echocardiography screening programmes. The sampling procedure and data collection details of the first study have been described in a previous study.^15^ Briefly, from January to December 2021, a multistage stratified random sampling method was adopted to select residents aged 35 years and older as study subjects (residence altitude range: 818–2242 m). 2500 m is considered to be the high altitude cut-off point for medical research.^12^ To further address the gap in epidemiological data on VHD among people living in the highland areas, we therefore conducted a second cross-sectional study from September 2023 to January 2024. The detailed sampling process has been provided in the online supplemental eMethods.

**Figure 1:**
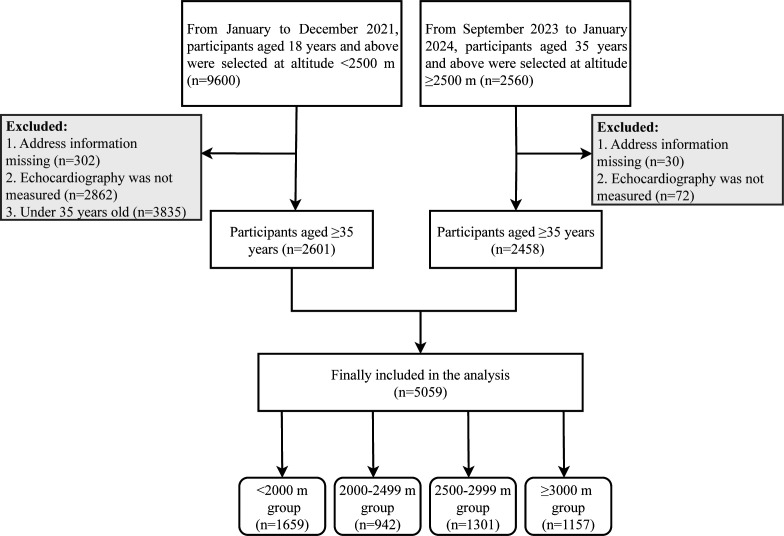
Flow chart of participant inclusion.

Exclusion criteria for the sample include under 35 years of age, missing address information and echocardiogram not being measured. 5059 participants were included in the final analyses. The sample inclusion process is shown in figure 1. All participants provided a written informed consent form.

### Altitude assessment

Altitude data in some previous studies were based on the county level.^16^ However, there is often a large variation in the altitude of residents living within the same county. The altitude of the township government in China is located in an easily accessible place for the vast majority of residents, so the altitude of the township government is well represented as their residential altitude.^17^ The altitude information was provided by the Yunnan Provincial Map Institute. Referring to previous studies^17^ and the distribution of participants’ altitude in this study, we classified the altitude into four categories: <2000 m, 2000–2499 m, 2500–2999 m and ≥3000 m.

### Data collection and measurement

Both two sequential cross-sectional studies used the same survey protocol and data collection forms. A survey team of approximately 20 trained physicians, echocardiologists, nurses, pharmacists and volunteers conducted questionnaires, physical examinations, echocardiographic measurements and laboratory tests for participants. First, participants’ demographic characteristics, behavioural lifestyle (smoking, alcohol consumption) and disease history were collected using a standardised questionnaire. Han Chinese is the dominant ethnic group in China, and we classified the ethnic variables as Han, Tibetans, Naxi and other ethnic minorities. Annual household income levels were classified as <¥20 000 or ≥¥20 000. Current smokers were defined as those who smoked one cigarette per day for more than 6 months, and current drinkers were those who drank alcohol at least once a week.^15^ Abdominal obesity was defined as a waist circumference of ≥90 cm for men and ≥85 cm for women.

Participants’ blood pressure was measured using an Omron HBP-1300 electronic sphygmomanometer. Hypertension was defined as systolic blood pressure (SBP) ≥140 mm Hg and/or diastolic blood pressure ≥90 mm Hg, or a previous diagnosis of hypertension.^15 16^ Finally, blood samples were collected from participants who had been fasting for more than 8 hours, and lipids and fasting blood glucose were measured.

### Echocardiography assessment

Detailed echocardiographic measurement procedures, quality control and variable definitions are provided in the online supplemental online supplemental eMethods. Stenotic and regurgitant VHD were assessed using semiquantitative and quantitative methods recommended by the American Society of Echocardiography Guidelines.^18^ AR severity was classified according to the ratio of regurgitant bundle width to LV outflow tract width as mild regurgitation: <25%; moderate: 25–64%; and severe: ≥65%, or according to the vena contracta width as mild: <0.3 cm; moderate: 0.3–0.6 cm; and severe: >0.6 cm. MR and tricuspid regurgitation (TR) severity were classified according to the ratio of regurgitant bundle area to atrial area as mild regurgitation: <20%; moderate: 21–40%; and severe: >40%, or according to the vena contracta width as mild: <0.3 cm; moderate: 0.3–0.69 cm; and severe: ≥0.7 cm.

The severity of valvular stenosis depends on the valve area and the mean pressure gradient of the restrictive orifice.^19 20^ Aortic stenosis (AS) was defined as mild with a valve jet velocity of 2.6-2.9 m/s, moderate with 3.0-4.0 m/s, and severe with ≥4.0 m/s. According to mitral valve area (<1.5 cm^2^, 1.0–1.5 cm^2^, <1.0 cm^2^), mitral stenosis (MS) was classified as mild, moderate or severe. Clinically significant VHD was defined as MS or AS, moderate or severe MR, AR, TR.^5 6^

### Statistical analysis

Participants’ characteristics were described according to four altitude groups. Continuous variables were presented as mean±SD and analysis of variance was used to compare differences. Categorical variables were reported as numbers and percentages (%), and comparisons between groups were performed using the χ^2^ test. We reported the prevalence of mild and clinically significant VHD and the proportions of VHD aetiology in different altitude groups.

Previous studies have shown that when the prevalence of an event is not rare in cross-sectional studies, it is more appropriate to report the prevalence ratio (PR) than the OR.^21^ To assess the association of different altitude groups with VHD, we constructed three multivariable Poisson regression models with robust variance to estimate PRs and 95% CIs, and stratified analyses according to the three major regurgitant VHDs (MR, TR, AR). Model adjustment details and multicollinearity diagnosis can be found in the online supplemental eMethods and table S1. As rare events of MS and AS were identified, we only reported the prevalence and number of patients and did not conduct the corresponding multivariable analyses. Statistical analyses were performed using R software V.4.3.2. A two-sided p<0.05 was considered statistically significant.

## Results

### Demographic and clinical characteristics

The characteristics of the participants are presented in table 1. Overall, a total of 5059 participants aged 35 years and above were included in this study, of which 2276 (45.0%) were males with a mean age of 55.7±11.9 years. 1858 participants were identified with at least mild VHD (36.7%), and patients with VHD were significantly older, predominantly female and minority, with lower income and education level, and with significant left atrial (LA) enlargement and pulmonary hypertension (all p<0.05). LA anteroposterior diameter and LV end-diastolic volume were higher in the VHD group.

**Table 1:** Characteristics of participants with and without VHD

	Total (n=5059)	VHD (n=1858)	No VHD (n=3201)	P value
**Demographics**				
Sex, male	2276 (45.0)	638 (34.3)	1638 (51.2)	<0.001
Age (years)	55.7±11.9	58.8±11.9	53.9±11.5	<0.001
Rural residence	2117 (41.8)	805 (43.3)	1312 (41.0)	0.104
Ethnicity				<0.001
Han	2431 (48.1)	866 (46.6)	1565 (48.9)	
Tibetan	1050 (20.8)	473 (25.5)	577 (18.0)	
Naxi	1068 (21.1)	364 (19.6)	704 (22.0)	
Other minorities	510 (10.0)	155 (8.3)	355 (11.1)	
Education attainment				<0.001
Illiteracy	1081 (21.4)	522 (28.1)	559 (17.5)	
Primary school	1941 (38.4)	757 (40.7)	1184 (37.0)	
Junior high school	1162 (23.0)	349 (18.8)	813 (25.4)	
High school and above	857 (17.2)	230 (12.4)	645 (20.1)	
Annual household income				<0.001
<¥20 000	2967 (58.6)	1191 (64.1)	1776 (55.5)	
≥¥20 000	2092 (41.4)	667 (35.9)	1425 (44.5)	
**Lifestyle and metabolic factors**				
Current smokers	1226 (24.2)	277 (14.9)	949 (29.6)	<0.001
Current drinkers	700 (13.8)	208 (11.2)	492 (15.4)	<0.001
Abdominal obesity	1717 (33.9)	560 (30.1)	1157 (36.1)	<0.001
FBG, mmol/L	5.3±1.7	5.1±1.4	5.5±1.8	<0.001
TC, mmol/L	5.0±1.1	5.0±1.1	5.0±1.1	0.131
SBP, mm Hg	136.5±20.8	137.6±21.6	135.8±20.3	0.003
DBP, mm Hg	82.9±12.3	81.3±12.1	83.8±12.4	0.002
**Echocardiography**				
LA-ap, mm	32.0±4.4	32.5±4.8	31.7±4.2	<0.001
LVEDD, mm	45.0±4.8	45.0±5.0	45.0±4.7	0.968
IVSd, mm	8.8±1.5	8.7±1.6	8.8±1.4	0.026
LVEDV, mL	98.0±30.5	101.3±34.1	96.0±28.0	<0.001
LVPWd, mm	8.5±1.3	8.4±1.3	8.5±1.3	0.044
LVESV, mL	35.2±14.4	36.9±16.0	34.3±13.3	<0.001
**LA enlargement**	650 (12.8)	407 (21.9)	243 (7.6)	<0.001
**PAH**	131 (2.6)	105 (5.7)	26 (0.8)	<0.001

Data are presented as mean±SD for continuous variables; categorical variables were reported as numbers and percentages (%).

DBP, diastolic blood pressure; FBG, fasting blood glucose; IVSd, interventricular septal thickness; LA, left atrial; LA-ap, left atrial anteroposterior diameter; LVEDD, left ventricular end-diastolic internal dimension; LVEDV, left ventricular end-diastolic volume; LVESV, left ventricular end-systolic volume; LVPWd, left ventricular posterior wall thickness in diastole; PAH, pulmonary arterial hypertension; SBP, systolic blood pressure; TC, total cholesterol; VHD, valvular heart disease.

Participants living at ≥3000 m altitude were more likely to be Tibetan (90.5%) and illiterate (37.4%), have a higher proportion of abdominal obesity (41.0%), as well as higher SBP (all p<0.001, online supplemental table S2). The prevalence of LA enlargement was found to increase with altitude, the proportion of pulmonary hypertension was similarly higher in the high-altitude group (online supplemental table S2).

### Prevalence of VHD

**Figure 2:**
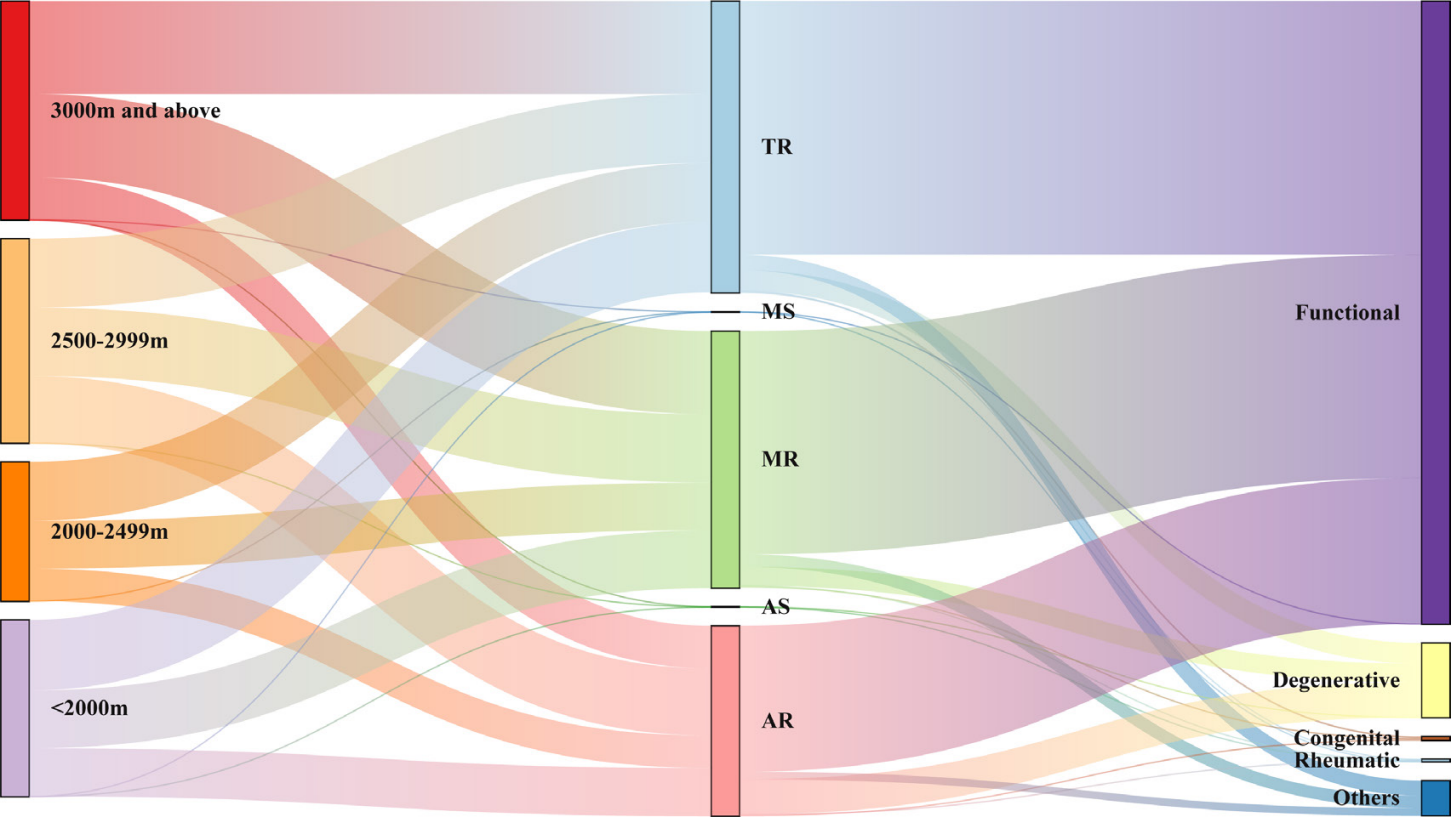
Valvular heart disease (VHD) and aetiological flow at different altitude groups. The Sankey diagram was applied to visualise the distribution of VHD and aetiological composition in different altitude groups. The first column of bars indicates the altitude groups, the second column indicates the various VHD subtypes and the third column of bars indicates the aetiology. This Sankey diagram represents the composition of the VHD subtypes in different altitude groups from left to right, and further flows into the aetiology of VHD. AR, aortic regurgitation; AS, aortic stenosis; MR, mitral regurgitation; MS, mitral stenosis; TR, tricuspid regurgitation.

Table 2 and figure 2 presented altitudinal differences in the prevalence of VHD and its subtypes. Among 1858 patients with mild and above VHD, TR was the most common VHD (24.5%), followed by MR (21.6%). Meanwhile, there were 129 patients (2.5%) with clinically significant VHD, and AR was the most prevalent (95/129, 73.6%).

**Table 2:** Prevalence of VHD and subtypes in different altitude groups

	Total (n=5059)	<2000 m (n=1659)	2000–2499 m (n=942)	2500–2999 m (n=1301)	≥3000 m (n=1157)
Any VHD	1858 (36.7)	505 (30.4)	385 (40.9)	456 (35.0)	512 (44.3)
MR					
None	3965 (78.4)	1413 (85.2)	739 (78.5)	1010 (77.6)	803 (69.4)
Mild	1062 (21.0)	237 (14.3)	200 (21.2)	280 (21.5)	345 (29.8)
Significant	32 (0.6)	9 (0.5)	3 (0.3)	11 (0.9)	9 (0.8)
TR					
None	3820 (75.5)	1359 (81.9)	690 (73.3)	1009 (77.6)	762 (65.9)
Mild	1205 (23.8)	293 (17.7)	251 (26.6)	281 (21.6)	380 (32.8)
Significant	34 (0.7)	7 (0.4)	1 (0.1)	11 (0.8)	15 (1.3)
AR					
None	4249 (84.0)	1454 (87.6)	804 (85.4)	1015 (78.0)	976 (84.4)
Mild	715 (14.1)	169 (10.2)	122 (13.0)	248 (19.1)	176 (15.2)
Significant	95 (1.9)	36 (2.2)	16 (1.7)	38 (2.9)	5 (0.4)
AS					
Significant	3 (0.1)	1 (0.1)	0 (0.0)	1 (0.1)	1 (0.1)
MS					
Significant	4 (0.1)	1 (0.1)	2 (0.2)	0 (0.0)	1 (0.1)

Data are presented as numbers and percentages (%).

AR, aortic regurgitation; AS, aortic stenosis; MR, mitral regurgitation; MS, mitral stenosis; TR, tricuspid regurgitation; VHD, valvular heart disease.

**Figure 3:**
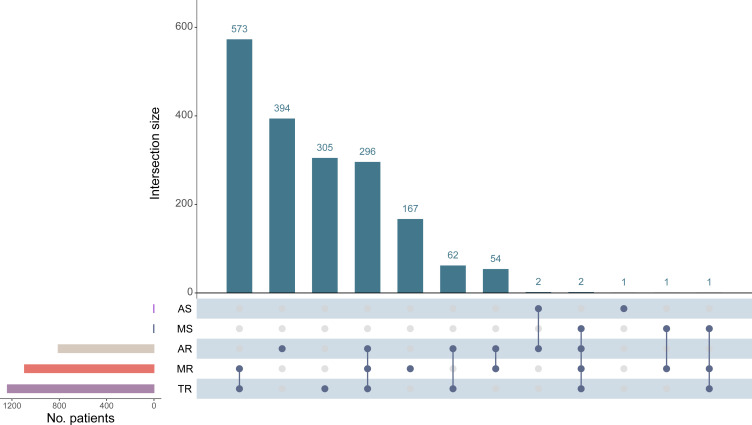
Upset diagram on distribution of single valvular heart disease (VHD) and multiple VHD. Because individuals often present with multiple valvular lesions, the Upset plot with R package ‘UpSetR’ was employed to present the distribution of comorbidities of VHD subtypes. The bar chart on the left side shows the number of cases in a single VHD. For the lower part, the presence of intersections between VHD subtypes is indicated by the vertical realisation of point-to-point connections. Solid lines are connected to indicate the presence of multiple VHD. The upper blue bar chart indicates the number of cases corresponding to the vertical intersection. For example, set 1 (the first vertical bar chart) indicates the number of patients with MR and TR in common, and set 2 indicates the number of patients after removing the intersection. AR, aortic regurgitation; AS, aortic stenosis; MR, mitral regurgitation; MS, mitral stenosis; TR, tricuspid regurgitation.

For all three major regurgitant VHDs (TR, MR, AR), the prevalence was higher in females than males (online supplemental table S3). After stratification by altitude, the prevalence of any VHD among participants in the <2000 m, 2000–2499 m, 2500–2999 m and ≥3000 m groups was 30.4%, 40.9%, 35.0% and 44.3%, respectively (table 2). Figure 3 presented the distribution of aggregated multiple VHD subtypes. 991 participants had multiple VHD (19.6%), with ‘MR+TR’ being the most prevalent (n=573).

### Altitude and risk of VHD

The fully adjusted model 3 showed that the PRs for VHD in the 2000–2499 m, 2500–2999 m and ≥3000 m groups were 1.28 (95% CI 1.15 to 1.42), 1.20 (95% CI 1.02 to 1.41) and 1.34 (95% CI 1.04 to 1.72) (table 3). A per 100 m increase in altitude was associated with a 3% heightened risk of VHD prevalence (PR=1.030, 95% CI 1.020 to 1.039, see online supplemental table S4). The restricted cubic spline curve unveiled a non-linear association between residential altitude and VHD (non-linear p<0.001) (online supplemental figure S3). Furthermore, we performed multivariable analysis with MR, TR and AR as dependent variables, and similar trends were observed (both p<0.05, online supplemental table S4). The results of the subgroup analyses showed that the PR for the association of altitude with VHD risk was significantly greater in men, ethnic minorities, high SBP and those aged ≥60 years (see online supplemental table S5).

**Table 3:** Multivariable Poisson regression models for residential altitude and risk of VHD

	Model 1		Model 2		Model 3	
	PR (95% CI)	P value	PR (95% CI)	P value	PR (95% CI)	P value
Any VHD						
<2000 m	1 (reference)		1 (reference)		1 (reference)	
2000–2499 m	1.31 (1.18 to 1.45)	<0.001	1.32 (1.19 to 1.47)	0.023	1.28 (1.15 to 1.42)	<0.001
2500–2999 m	1.29 (1.11 to 1.51)	0.001	1.27 (1.07 to 1.50)	0.005	1.20 (1.02 to 1.41)	0.030
≥3000 m	1.36 (1.05 to 1.75)	0.020	1.36 (1.04 to 1.77)	<0.001	1.34 (1.04 to 1.72)	0.023
P for trend	<0.001		<0.001		<0.001	
MR						
<2000 m	1 (reference)		1 (reference)		1 (reference)	
2000–2499 m	1.50 (1.26 to 1.79)	<0.001	1.43 (1.21 to 1.69)	<0.001	1.38 (1.19 to 1.60)	<0.001
2500–2999 m	1.53 (1.22 to 1.92)	<0.001	1.25 (1.04 to 1.51)	0.017	1.34 (1.03 to 1.74)	0.029
≥3000 m	1.65 (1.13 to 2.40)	0.009	1.43 (1.21 to 1.70)	<0.001	1.52 (1.09 to 2.13)	0.015
P for trend	<0.001		<0.001		<0.001	
TR						
<2000 m	1 (reference)		1 (reference)		1 (reference)	
2000–2499 m	1.47 (1.26 to 1.71)	<0.001	1.38 (1.19 to 1.61)	<0.001	1.38 (1.19 to 1.60)	<0.001
2500–2999 m	1.34 (1.08 to 1.66)	0.007	1.37 (1.05 to 1.78)	0.018	1.08 (0.92 to 1.27)	0.352
≥3000 m	1.61 (1.15 to 2.25)	0.005	1.53 (1.09 to 2.15)	0.013	1.42 (1.22 to 1.64)	<0.001
P for trend	<0.001		<0.001		0.001	
AR						
<2000 m	1 (reference)		1 (reference)		1 (reference)	
2000–2499 m	1.20 (0.98 to 1.47)	0.071	1.27 (1.04 to 1.56)	0.018	1.27 (1.04 to 1.55)	0.020
2500–2999 m	1.96 (1.54 to 2.49)	<0.001	2.22 (1.79 to 2.74)	<0.001	2.38 (1.92 to 2.95)	<0.001
≥3000 m	1.84 (1.20 to 2.83)	0.005	1.26 (1.01 to 1.56)	0.041	1.33 (1.06 to 1.67)	0.012
P for trend	<0.001		<0.001		<0.001	

Model 1 adjusted for sex, age, residence, ethnicity (Han Chinese or ethnic minority), education level and annual household income.

Model 2 was further adjusted for lifestyle and metabolic factors (including current smokers, current drinkers, abdominal obesity, FBG, TC and blood pressure).

Model 3 was further adjusted for echocardiographic measurement variables (LA-ap, LVEDD, IVSd, LVEDV, LVPWd, LVESV and PAH) based on model 2.

AR, aortic regurgitation; FBG, fasting blood glucose; IVSd, interventricular septal thickness; LA-ap, left atrial anteroposterior diameter; LVEDD, left ventricular end-diastolic internal dimension; LVEDV, left ventricular end-diastolic volume; LVESV, left ventricular end-systolic volume; LVPWd, left ventricular posterior wall thickness in diastole; MR, mitral regurgitation; PAH, pulmonary arterial hypertension; PR, prevalence ratio; TC, total cholesterol; TR, tricuspid regurgitation; VHD, valvular heart disease.

### Aetiology of VHD

Figures2  4 demonstrated the distribution of VHD aetiologies among participants in different altitude groups. Overall, regardless of altitude subgroup, the most predominant aetiology of mild and above VHD was functional (85.4%), followed by degenerative (9.0%). Among functional regurgitant VHD, TR is the main subtype, followed by MR.

**Figure 4:**
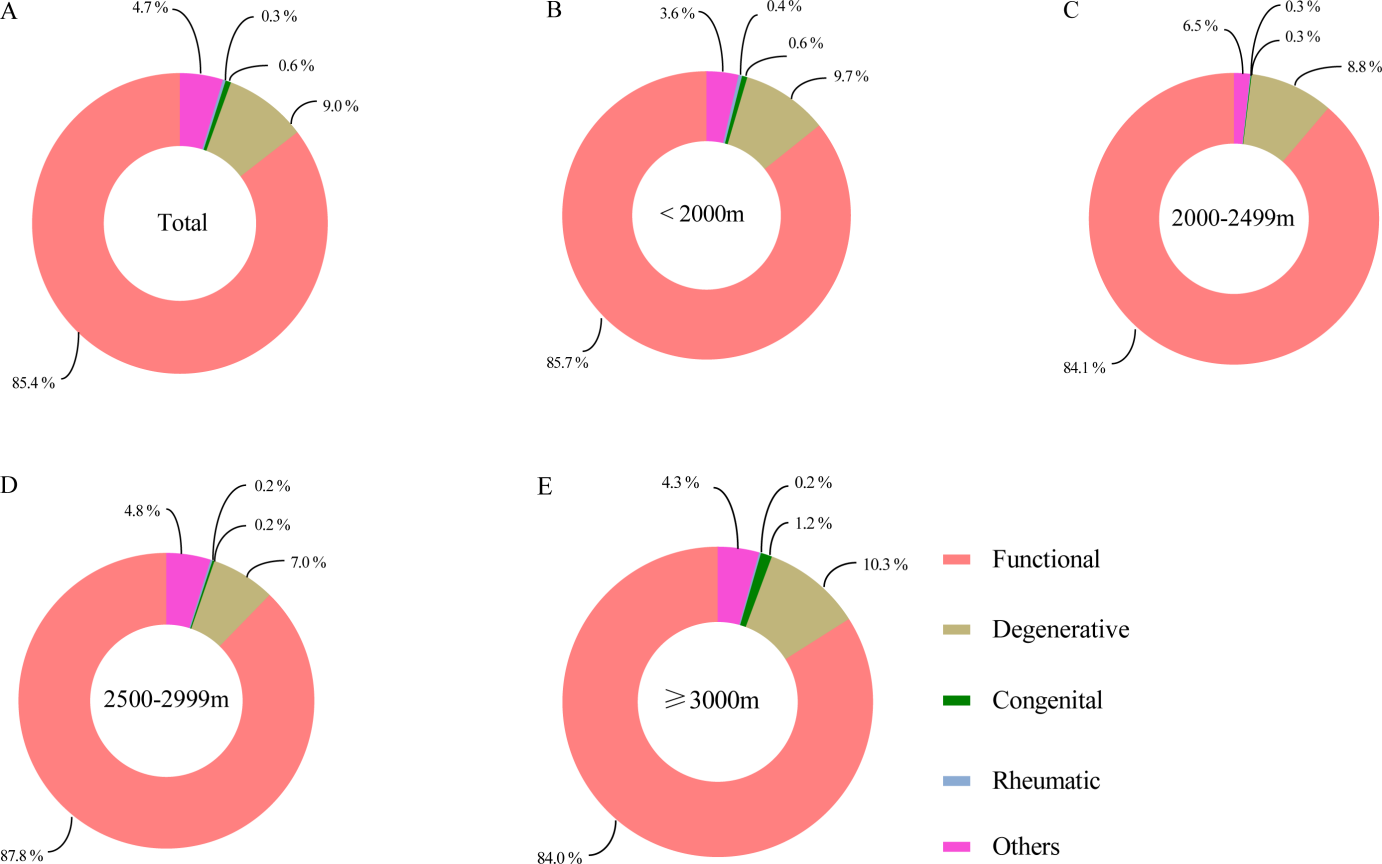
Aetiology distribution of mild and above valvular heart disease (VHD) in different altitude groups. (A) Distribution of aetiology for all participants; (B) participants at altitudes < 2000 m; (C) participants at altitudes 2000-2499 m; (D) participants at altitudes 2500-2999 m; (E) participants at altitudes ≥3000 m.

Further, we restricted the analysis to the aetiological composition of the 129 patients with clinically significant VHD (online supplemental table S6 and figure S2), degenerative VHD was the most common (54.2%). However, degenerative remains the major aetiology among patients with significant VHD in the <3000 m altitude, whereas 84.2% of patients were identified as functional in the ≥3000 m altitude. When stratified according to the valve lesion area, functional was the most common aetiology of MR (84.4%) and TR (100.0%) (online supplemental table S7).

## Discussion

This community-based echocardiography screening study provides the first comprehensive profile of the VHD characteristics, spectrum and aetiology differences at different altitudes in Yunnan Province, China. Our study revealed for the first time that the prevalence of mild and above VHD in this region was 36.7%, with TR as the most common VHD (24.5%), followed by MR (21.6%) and AR (16.0%). Similar to previous findings in African Americans, TR was the predominant regurgitant VHD (17.2%).^22^ Although most VHD was identified as mild in this study, mild VHD has the potential to progress to severe.^23^ Given the high burden of VHD in China and globally, our findings suggest that there is an urgent need to provide public and healthcare professionals with health awareness on VHD, early screening for the pathogenic factors of VHD to reduce adverse clinical outcomes.

A cohort study confirmed a linear trend between the severity of aortic valve lesions and the risk of myocardial infarction.^24^ Our study found that AR was the predominant clinically significant VHD, and therefore more healthcare resources and research should focus on the types of VHD that are becoming increasingly prevalent or have a poorer prognosis. Meanwhile, we found that the prevalence of clinically significant VHD was 2.5%, which is close to that of the US population (2.5%),^5^ but slightly lower than the national level of China in 2012–2015 (3.8%)^7^ and the US study (3.1%).^25^

It is estimated that 500.3 million people live in areas ≥1500 m above sea level, 81.6 million people live in areas ≥2500 m altitude and almost all of these areas belong to low- and middle-income countries.^10^ VHD is a rapidly growing cause of cardiovascular morbidity and mortality globally,^26^ so it is critical to understand trends in the geographical distribution. A study from Turkey reported a significantly higher prevalence of mitral valve prolapse at moderate altitude than at sea level (6.2% vs 2.0%).^27^ To our knowledge, there are no epidemiological and aetiological studies of VHD based on residential altitude stratification. In this study, the risk of VHD prevalence increased at high altitude with a non-linear increasing trend (p for non-linear <0.001). The altitudinal differences in VHD prevalence in our study may contribute to the complex interaction of low atmospheric pressure, partial pressure of oxygen, climate, individual genetics, lifestyle and socioeconomic factors at high altitude.^12^ Consistent with studies from Denmark^28^ and UK^6^ that have shown low socioeconomic status is associated with VHD and comorbidities, our study also found most of the residents in high-altitude areas are ethnic minorities (Tibetan and Naxi ethnic groups), with lower socioeconomic status, which may have a significant impact on the association of altitude and increased VHD prevalence.

Several previous studies revealed that SBP is associated with an increased risk of developing VHD.^26 29 30^ Our study by subgroup analysis found that those participants who lived at high altitude and had high SBP (≥140 mm Hg) had a significantly higher risk of VHD than those with low SBP (p for interaction=0.008, see online supplemental table S5). In addition, progressive LV remodelling due to hypertension may ultimately lead to geometric distortion of multiple elements of the mitral valve apparatus and subsequent functional MR,^22^ while hypertension is the main risk factor for AR.^30^

Actually, this study found that functionality is the predominant aetiology in the vast majority of patients with mild and above VHD. Consistent with a previous study conducted among 38 841 patients with VHD in China,^23^ degenerative VHD was the predominant aetiology than functional (54.2% vs 45.8%) when we analysed 129 patients with clinically significant VHD. We found altitudinal differences in the aetiology of clinically significant VHD, with degenerative VHD predominating in the <3000 m groups. In recent decades, degenerative VHD has replaced rheumatic as the leading aetiology of VHD,^23^ although a large-scale survey in China still found rheumatic VHD to be the most prevalent, with degenerative being the second.^7^ Early detection and treatment can avoid and delay the onset of heart failure.^7^ Given the large population at high altitude, with limited medical resources and poor economic level, and the high prevalence of VHD, there is an urgent need to implement effective VHD risk factor control strategies and promote public health awareness.

### Study limitations

Some potential limitations of this study should be considered. First, since this study identified a number of patients with mild valvular regurgitation, it is difficult to determine the aetiology of this early lesion. Second, the non-linear relationship between altitude and VHD observed in this cross-sectional study may reflect the influence of unmeasured confounding factors, such as low-density lipoprotein-cholesterol, which have been shown to influence VHD.^31^ While our analysis controlled for several covariates, the possibility of residual confounding cannot be ruled out. Third, LA volume may more accurately define LA enlargement compared with LA diameter; moreover, we did not obtain data on LV ejection fraction, thus limiting the aetiology interpretation of MR. Finally, although we attempted to control for confounders in the design and implementation of this study, the COVID-19 pandemic may inevitably have had some impact on the findings.

## Conclusions

This study innovatively revealed the prevalence, spectrum, aetiology and risk of VHD at different altitudes based on community echocardiographic screening. We found a significantly higher prevalence of VHD in people living at high altitude areas; we also identified patients with clinically significant VHD living at <3000 m altitude attributed primarily to degenerative, whereas functional VHD was most prevalent in those residing at ≥3000 m altitude. Given that the majority of populations living in low- and middle-income countries reside at higher altitudes, our study could provide perspective insights into early screening for VHD and improve health awareness among healthcare practitioners and public education in these regions with limited medical resources.

## Supplementary material

10.1136/heartjnl-2024-325221 online supplemental file 1

## Data Availability

Data are available upon reasonable request.
