# Supplementary material for: Prevalence, spectrum and aetiology of valvular heart disease based on community echocardiographic screening transition from different altitudes in Yunnan, China
Source: Heart. 2025 Mar 3;111(22):e325221. doi: 10.1136/heartjnl-2024-325221 (PMC12573351; doi:10.1136/heartjnl-2024-325221)
Supplement: online supplemental file 1 [file heartjnl-111-22-s001.doc]

**Prevalence, spectrum, and aetiology of valvular heart disease based on community echocardiographic screening- transition from different altitudes in Yunnan, China**

**Supplementary Material**

[eMethods 2](#__RefHeading___Toc191035167)

[Figure S1. Location of the sampling sites 5](#__RefHeading___Toc191035168)

[Figure S2. Etiology distribution of clinically significant VHD in different altitude groups (n=129) 5](#__RefHeading___Toc191035169)

[Figure S3. Non-linear association between altitude and overall VHD, MR, TR, and AR by restricted cubic spline curves 6](#__RefHeading___Toc191035170)

[Table S1. Diagnostic of model multicollinearity 7](#__RefHeading___Toc191035171)

[Table S2. Characteristics of participants in different altitude groups 8](#__RefHeading___Toc191035172)

[Table S3. Characteristics of participants with regurgitant valvular disease 9](#__RefHeading___Toc191035173)

[Tabel S4. Multivariable Poisson regression models for residential altitude and risk of VHD 10](#__RefHeading___Toc191035174)

[Table S5. Subgroup analyses of the association between altitude and VHD according to sex, age, ethnic group, and SBP 11](#__RefHeading___Toc191035175)

[Table S6. Etiological distribution of 129 patients with clinically significant VHD 13](#__RefHeading___Toc191035176)

[Table S7. Etiological distribution of patients with clinically significant MR and TR 13](#__RefHeading___Toc191035177)

[Reference 14](#__RefHeading___Toc191035178)

# eMethods

**Study design and participants**

For the first investigation, a multistage stratified random sampling method was conducted to invite participants aged 18 years and older from eight counties in the middle and low altitude regions of Yunnan Province (n=9600) and to conduct a cardiovascular disease screening project. Because the prevalence of Valvular heart disease (VHD) is very low in people under 35 years old, and to be comparable with other studies, we included permanent residents aged ≥35 years as study subjects.

For the second investigation, firstly, all townships in Yunnan Province above 2500 m altitude were divided into rural and urban, and 2 townships were selected in each of these two strata by simple random sampling method. Secondly, 4 village committees continued to be randomly selected from the 4 townships sampled above, with a total of 16 village committees. Finally, residents aged 35 years and above who have been living in the sampled village committees for more than 6 months were included as participants in the survey for this screening project.

**Echocardiography assessment**

Transthoracic echocardiographic assessment of each participant was performed at the screening center by two experienced and trained echocardiographers from a specialist cardiovascular hospital (Fuwai Yunnan Hospital, Chinese Academy of Medical Sciences) using a portable ultrasound device (Vivid™ iq, GE Medical Systems Trade and Development Co., Ltd.) with Probe (M5Sc). The echocardiography device is equipped with M-mode, 2D, pulsed wave Doppler, continuous wave Doppler, and color flow Doppler modes.

The structure, function, location, and number of heart valves, valve thickening, echo enhancement, calcification, and flow velocity of each valve were observed in the subjects and all the investigation data and echocardiographic images were stored for further offline evaluation. These ultrasound images were re-verified for diagnosis by a third sonographer before the statistical analysis to ensure the accuracy of the diagnostic results.

According to the ASE criteria ^1^, the following echocardiographic indicators were also collected: left atrial anteroposterior diameter (LA-ap), left ventricular (LV) end-diastolic internal dimension (LVEDD), interventricular septal thickness (IVSd), LV end-diastolic volume (LVEDV), LV posterior wall thickness in diastole (LVPWd), LV end-systolic volume (LVESV). In addition, the sonographer measured tissue Doppler-derived velocities of the tricuspid regurgitant jet and used the simplified Bernoulli’s equation to estimate the systolic right ventricle-to-atrium pressure gradient. For pulmonary artery systolic pressure (PASP) the estimated equation is: PASP = 4 × (velocity of the tricuspid regurgitant jet) +estimated right atrial pressure. Pulmonary arterial hypertension (PAH) was defined as PASP >35 mm Hg.^2^ Given that this study was a community-based screening program, although this method is not the gold standard for the diagnosis of PAH, it is a readily available bedside technique and non-invasive and is recognized as the primary non-invasive method for PAH assessment.^2-3^ The etiology of VHD was determined based on echocardiography and the participant's medical history and clinical background and was classified as degenerative, rheumatic, functional, or congenital.^4-5^ Based on the current data, some VHD patients with unclear etiology were classified as ‘other’. ^5^ Left atrial enlargement was identified as left atrial diameter exceeding 40 mm in men and 38 mm in women.^6-8^

**Model fitting steps**

We developed a 3-model analysis protocol by adding covariates. Model 1 adjusted for sex, age, residence, ethnicity (Han Chinese or ethnic minority), education level, and annual household income). Model 2 was further adjusted for lifestyle and metabolic factors (including current smokers, current drinkers, abdominal obesity, FBG, TC, and blood pressure). Model 3 was further adjusted for echocardiographic measurement variables (LA-ap, LVEDD, IVSd, LVEDV, LVPWd, LVESV, and PAH) based on Model 2. Since the “ethnicity” variable was discretely (unbalanced) distributed across altitude groups (see Table S2), we constructed the multivariate models using binary variables (Han Chinese or ethnic minority).

All multivariable analysis models were performed using the <2000 m altitude group as a reference. In addition, we tested for linear trends by including the median value of each altitude group as a continuous variable in the models using multivariable Poisson regression models with robust variance.

Finally, altitude was considered as a continuous variable ^7^, and a restricted cubic spline function (4 knots) with logistic regression model was employed to explore the non-linear association between altitude increase and the prevalence of VHD.

**Diagnostic of model multicollinearity**

We tested the problem of multicollinearity in Model 3 using linear regression, and the following table gives the VIF and tolerance values for altitude and ethnicity (Table S1).


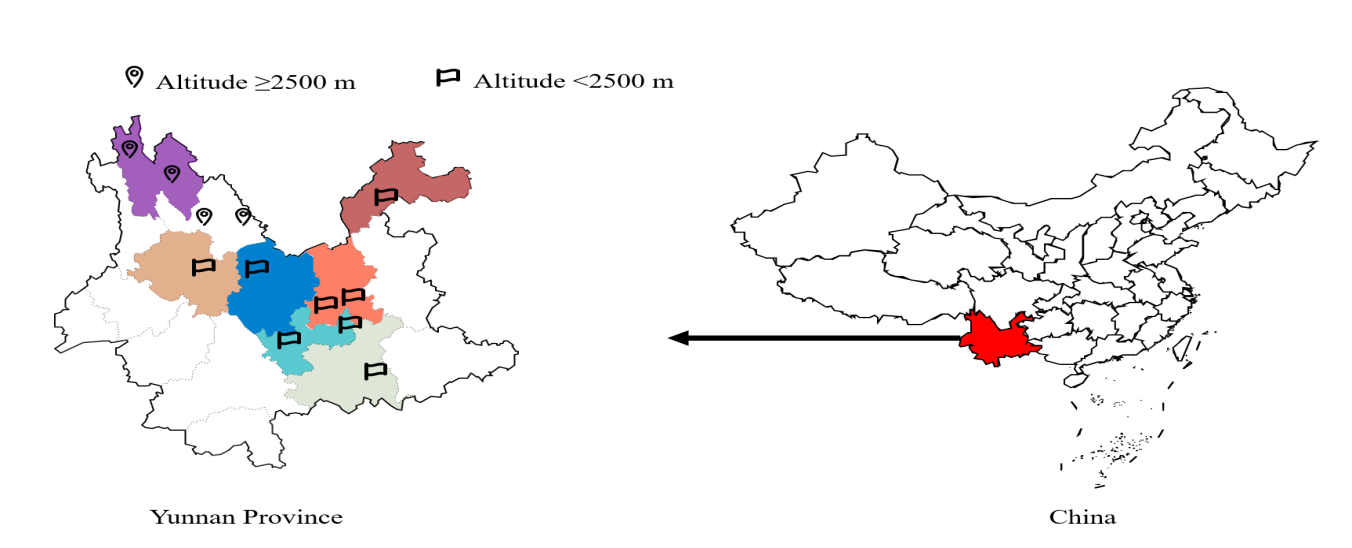


# Figure S1. Location of the sampling sites


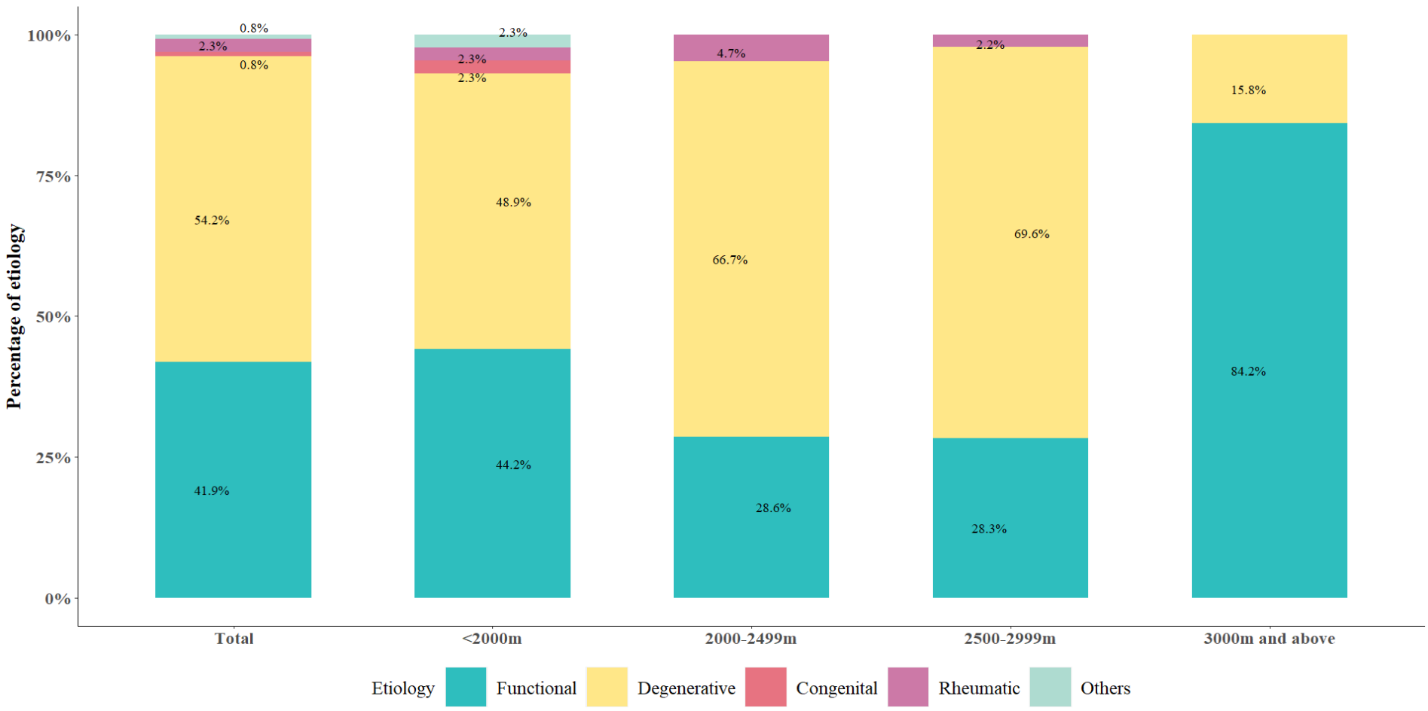


# Figure S2. Etiology distribution of clinically significant VHD in different altitude groups (n=129)


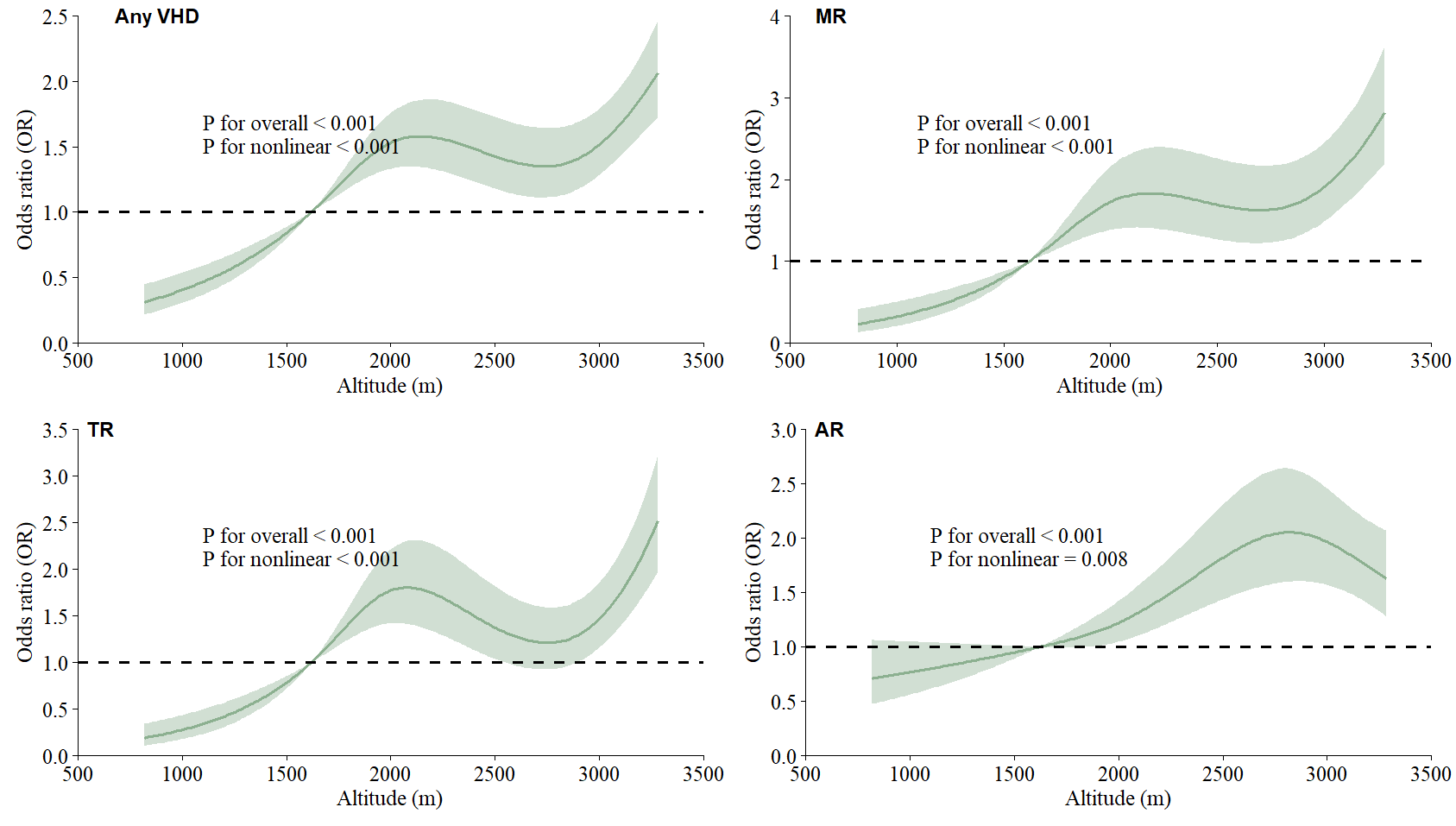


# Figure S3. Non-linear association between altitude and overall VHD, MR, TR, and AR by restricted cubic spline curves

Note: VHD, valvular heart disease; MR, mitral regurgitation; TR, tricuspid regurgitation; AR, aortic regurgitation.

# Table S1. Diagnostic of model multicollinearity

|  | **Collinearity Statistics** | |
| --- | --- | --- |
|  | **VIF** | **Tolerance** |
| **Multivariable without dummy variable conversion** |  |  |
| Altitude | 2.642 | 0.379 |
| Ethnicity | 2.526 | 0.960 |
| **Dummy variable conversion** |  |  |
| Altitude |  |  |
| 2000-2499 m | 1.436 | 0.696 |
| 2500-2999 m | 3.737 | 0.268 |
| ≥3000 m | 3.411 | 0.293 |
| Ethnicity |  |  |
| Minority | 3.518 | 0.284 |

VIF: variance inflation factor.

# Table S2. Characteristics of participants in different altitude groups

|  | **Total**  **(n=5059)** | **<2000 m**  **(n=1659)** | **2000-2499 m**  **(n=942)** | **2500-2999 m**  **(n=1301)** | **≥3000 m**  **(n=1157)** | **P value** |
| --- | --- | --- | --- | --- | --- | --- |
| **Demographics** |  |  |  |  |  |  |
| **Male** | 2276 (45.0) | 836 (50.4) | 384 (40.8) | 622 (47.8) | 434 (37.5) | <0.001 |
| **Age (years)** | 55.7±11.9 | 54.8±13.2 | 56.3±13.2 | 54.9±10.1 | 57.3±10.2 | <0.001 |
| **Urban residence** | 2942 (58.2) | 880 (53.0) | 617 (65.5) | 789 (60.6) | 656 (56.7) | <0.001 |
| **Ethnicity** |  |  |  |  |  | <0.001 |
| Han | 2431 (48.1) | 1429 (86.1) | 881(93.5) | 76 (5.8) | 45 (3.9) |  |
| Tibetan | 1050 (20.8) | 0 (0.0) | 0 (0.0) | 3 (0.2) | 1047 (90.5) |  |
| Naxi | 1068 (21.1) | 0 (0.0) | 0 (0.0) | 1034 (79.5) | 34 (2.9) |  |
| Other minorities | 510 (10.0) | 230 (13.9) | 61 (6.5) | 188 (14.5) | 31 (2.7) |  |
| **Education attainment** |  |  |  |  |  | <0.001 |
| Illiteracy | 1081 (21.4) | 288 (17.4) | 118 (12.5) | 242 (18.6) | 433 (37.4) |  |
| Primary school | 1941 (38.4) | 509 (30.7) | 243 (25.8) | 665 (51.1) | 524 (45.3) |  |
| Junior high school | 1162 (23.0) | 478 (28.8) | 319 (33.9) | 260 (20.0) | 105 (9.1) |  |
| High school and above | 875 (17.2) | 384 (23.1) | 262 (27.8) | 134 (10.3) | 95 (8.2) |  |
| **Annual household income <20,000 yuan** | 2967 (58.6) | 579 (34.9) | 539 (57.2) | 1054 (81.0) | 795 (68.7) | <0.001 |
| **Lifestyle and metabolic factors** |  |  |  |  |  |  |
| Current smokers | 1226 (24.2) | 391 (23.6) | 206 (21.9) | 391 (30.1) | 238 (20.6) | <0.001 |
| Current drinkers | 700 (13.8) | 159 (9.6) | 110 (11.7) | 250 (19.2) | 181 (15.6) | <0.001 |
| Abdominal obesity | 1717 (33.9) | 502 (30.3) | 339 (36.0) | 402 (30.9) | 474 (41.0) | <0.001 |
| FBG, mmol/L | 5.3±1.7 | 5.4±1.5 | 5.4±1.7 | 5.3±1.7 | 5.1±1.8 | <0.001 |
| TC, mmol/L | 5.0±1.1 | 4.9±1.0 | 5.2±1.0 | 5.1±1.3 | 5.0±0.9 | <0.001 |
| SBP, mmHg | 136.5±20.9 | 132.6±19.3 | 132.3±19.3 | 134.0±20.0 | 136.2±20.4 | <0.001 |
| DBP, mmHg | 82.8±12.4 | 80.6±12.4 | 83.7±11.9 | 86.2±12.4 | 79.7±11.6 | <0.001 |
| **Echocardiography** |  |  |  |  |  |  |
| LA-ap, mm | 32.0±4.4 | 31.5±4.4 | 31.1±4.2 | 32.3±4.7 | 33.1±4.1 | <0.001 |
| LVEDD, mm | 45.0±4.8 | 44.3±4.9 | 45.1±4.7 | 44.8±4.8 | 46.0±4.7 | <0.001 |
| IVSd, mm | 8.8±1.5 | 9.1±1.5 | 9.1±1.6 | 8.3±1.4 | 8.4±1.3 | <0.001 |
| LVEDV, mL | 98.0±30.5 | 92.1±25.0 | 99.6±26.7 | 110.2±29.8 | 91.3±36.5 | <0.001 |
| LVPWd, mm | 8.5±1.3 | 8.8±1.4 | 8.5±1.3 | 8.2±1.1 | 8.3±1.2 | <0.001 |
| LVESV, mL | 35.2±14.4 | 30.7±11.8 | 29.4±12.4 | 45.3±15.5 | 35.1±12.0 | <0.001 |
| **LA enlargement** | 650 (12.8) | 78 (4.7) | 45 (4.8) | 231 (17.8) | 296 (25.6) | <0.001 |
| **PAH** | 131 (2.6) | 13 (0.8) | 11 (1.2) | 68 (5.2) | 39 (3.4) | <0.001 |

Note: Data are presented as mean± standard deviation for continuous variables, categorical variables were reported as numbers and percentages (%).

FBG, fasting blood glucose; PAH, pulmonary arterial hypertension; LA, left atrial; LV, left ventricular; LA-ap, Left atrial anteroposterior diameter; LVEDD, LV end-diastolic internal dimension; IVSd, Interventricular septal thickness; LVEDV, LV end-diastolic volume; LVPWd, LV posterior wall thickness in diastole; LVESV, LV end-systolic volume.

# Table S3. Characteristics of participants with regurgitant valvular disease

|  | **MR** | **TR** | **AR** |
| --- | --- | --- | --- |
| **Sex** |  |  |  |
| Male | 372 (16.3) | 417 (18.3) | 316 (13.9) |
| Female | 722 (25.9) | 822 (29.5) | 494 (17.8) |
| P value | <0.001 | <0.001 | <0.001 |
| **Age group (years)** |  |  |  |
| 35-44 | 153 (14.9) | 192 (18.7) | 45 (4.4) |
| 45-54 | 288 (20.1) | 310 (21.7) | 112 (7.8) |
| 55-64 | 306 (22.5) | 370 (27.3) | 237 (17.5) |
| 65-74 | 236 (27.3) | 263 (30.4) | 271 (31.4) |
| ≥75 | 111 (29.1) | 104 (27.3) | 145 (38.1) |
| P value | <0.001 | <0.001 | <0.001 |
| **Ethnicity** |  |  |  |
| Han | 435 (17.9) | 537 (22.1) | 347 (14.3) |
| Tibetan | 332 (31.6) | 368 (35.0) | 163 (15.5) |
| Naxi | 238 (22.3) | 238 (22.3) | 217 (20.3) |
| Other minorities | 89 (17.4) | 96 (18.8) | 83 (16.2) |
| P value | <0.001 | <0.001 | <0.001 |
| **Residence** |  |  |  |
| Urban | 630 (21.4) | 761 (25.9) | 402 (13.7) |
| Rural | 464 (21.9) | 478 (22.6) | 408 (19.3) |
| P value | 0.386 | 0.027 | <0.001 |

Note: Data are presented as number of cases (prevalence, %), prevalence = number of patients in each subgroup/number of samples in each subgroup. Comparisons between groups were performed using the Chi-square test.

MR, mitral regurgitation; TR, tricuspid regurgitation; AR, aortic regurgitation.

# Tabel S4. Multivariable Poisson regression models for residential altitude and risk of VHD

|  | **Model 1** | |  | **Model 2** | |  | **Model 3** | |
| --- | --- | --- | --- | --- | --- | --- | --- | --- |
|  | **PR, 95% CI** | **P value** |  | **PR, 95% CI** | **P value** |  | **PR, 95% CI** | **P value** |
| **Any VHD** |  |  |  |  |  |  |  |  |
| Per 100 m increment in altitude | 1.036  (1.027-1.045) | <0.001 |  | 1.033  (1.024-1.042) | <0.001 |  | 1.030  (1.020-1.039) | <0.001 |
| Per 1-SD increment in altitude (657.3m) | 1.260  (1.119-1.333) | <0.001 |  | 1.236  (1.167-1.310) | <0.001 |  | 1.211  (1.141-1.286) | <0.001 |
| **MR** |  |  |  |  |  |  |  |  |
| Per 100 m increment in altitude | 1.052  (1.039-1.065) | <0.001 |  | 1.048  (1.035-1.062) | <0.001 |  | 1.040  (1.026-1.054) | <0.001 |
| Per 1-SD increment in altitude (657.3m) | 1.395  (1.284-1.515) | <0.001 |  | 1.363  (1.252-1.485) | <0.001 |  | 1.295  (1.187-1.413) | <0.001 |
| **TR** |  |  |  |  |  |  |  |  |
| Per 100 m increment in altitude | 1.052  (1.040-1.064) | <0.001 |  | 1.047  (1.034-1.060) | <0.001 |  | 1.041  (1.028-1.054) | <0.001 |
| Per 1-SD increment in altitude (657.3m) | 1.397  (1.294-1.507) | <0.001 |  | 1.350  (1.247-1.462) | <0.001 |  | 1.300  (1.119-1.409) | <0.001 |
| **AR** |  |  |  |  |  |  |  |  |
| Per 100 m increment in altitude | 1.031  (1.017-1.046) | <0.001 |  | 1.033  (1.018-1.048) | <0.001 |  | 1.029  (1.014-1.045) | <0.001 |
| Per 1-SD increment in altitude (657.3m) | 1.224  (1.115-1.343) | <0.001 |  | 1.235  (1.122-1.359) | <0.001 |  | 1.208  (1.095-1.334) | <0.001 |

Model 1 adjusted for sex, age, residence, ethnicity (Han Chinese or ethnic minority), education level, and annual household income.

Model 2 was further adjusted for lifestyle and metabolic factors (including current smokers, current drinkers, abdominal obesity, FBG, TC, and blood pressure).

Model 3 was further adjusted for echocardiographic measurement variables (LA-ap, LVEDD, IVSd, LVEDV, LVPWd, LVESV, and PAH) based on Model 2.

Note: VHD, valvular heart disease; MR, mitral regurgitation; TR, tricuspid regurgitation; AR, aortic regurgitation; PR, prevalence ratio.

# Table S5. Subgroup analyses of the association between altitude and VHD according to sex, age, ethnic group, and SBP

|  | **Model 1** | |  | **Model 2** | |  | **Model 3** | | **P for interaction** |
| --- | --- | --- | --- | --- | --- | --- | --- | --- | --- |
|  | **PR, 95% CI** | **P value** |  | **PR, 95% CI** | **P value** |  | **PR, 95% CI** | **P value** |
| **Male** |  |  |  |  |  |  |  |  | <0.001 |
| <2000 m | 1 |  |  | 1 |  |  | 1 |  |  |
| 2000-2499 m | 0.899  (0.687-1.176) | 0.437 |  | 0.937 (0.737-1.215) | 0.625 |  | 0.959  (0.739-1.248) | 0.755 |  |
| 2500-2999 m | 2.059  (1.555-2.726) | <0.001 |  | 1.762  (1.322-2.348) | <0.001 |  | 1.497  (1.111-2.018) | 0.008 |  |
| ≥3000 m | 2.323  (1.747-3.088) | <0.001 |  | 1.890  (1.401-2.549) | <0.001 |  | 1.867  (1.377-2.531) | <0.001 |  |
| *P* for trend |  | <0.001 |  |  | <0.001 |  |  | <0.001 |  |
| **Female** |  |  |  |  |  |  |  |  |  |
| <2000 m | 1 |  |  | 1 |  |  | 1 |  |  |
| 2000-2499 m | 1.315  (0.176-1.470) | <0.001 |  | 1.349  (1.206-1.509) | <0.001 |  | 1.280  (1.114-1.431) | <0.001 |  |
| 2500-2999 m | 0.966  (0.808-1.156) | 0.706 |  | 1.034  (0.864-1.237) | 0.715 |  | 0.859  (0.710-1.039) | 0.116 |  |
| ≥3000 m | 1.196  (1.008-1.417) | 0.004 |  | 1.283  (1.082-1.522) | 0.004 |  | 1.159  (0.976-1.377) | 0.092 |  |
| *P* for trend |  | 0.001 |  |  | <0.001 |  |  | 0.003 |  |
| **Age, <60** |  |  |  |  |  |  |  |  | 0.025 |
| <2000 m | 1 |  |  | 1 |  |  | 1 |  |  |
| 2000-2499 m | 1.234  (1.067-1.427) | 0.005 |  | 1.285  (1.112-1.485) | 0.001 |  | 1.233  (1.067-1.425) | 0.004 |  |
| 2500-2999 m | 1.107  (0.904-1.356) | 0.326 |  | 1.067  (0.869-1.309) | 0.536 |  | 0.861  (0.694-1.069) | 0.176 |  |
| ≥3000 m | 1.336  (1.092-1.635) | 0.005 |  | 1.267  (1.031-1.557) | 0.024 |  | 1.183  (0.964-1.1452) | 0.108 |  |
| *P* for trend |  | <0.001 |  |  | <0.001 |  |  | 0.004 |  |
| **Age, ≥60** |  |  |  |  |  |  |  |  |  |
| <2000 m | 1 |  |  | 1 |  |  | 1 |  |  |
| 2000-2499 m | 1.379  (1.185-1.604) | <0.001 |  | 1.368  (1.178-1.588) | <0.001 |  | 1.324  (1.141-1.537) | <0.001 |  |
| 2500-2999 m | 1.540  (1.255-1.890) | <0.001 |  | 1.541  (1.256-1.890) | <0.001 |  | 1.288  (1.039-1.597) | 0.021 |  |
| ≥3000 m | 1.566  (1.275-1.923) | <0.001 |  | 1.598  (1.301-1.964) | <0.001 |  | 1.462  (1.187-1.800) | <0.001 |  |
| *P* for trend |  | <0.001 |  |  | <0.001 |  |  | <0.001 |  |
| **Han Chinese** |  |  |  |  |  |  |  |  | <0.001 |
| <2000 m | 1 |  |  | 1 |  |  | 1 |  |  |
| 2000-2499 m | 1.111  (0.998-1.237) | 0.054 |  | 1.148  (1.031-1.279) | 0.012 |  | 1.137  (1.018-1.270) | 0.023 |  |
| 2500-2999 m | 1.207  (0.911-1.597) | 0.190 |  | 1.112  (0.845-1.490) | 0.427 |  | 0.925  (0.680-1.256) | 0.617 |  |
| ≥3000 m | 0.746  (0.481-1.159) | 0.193 |  | 0.831  (0.538-1.283) | 0.403 |  | 0.776  (0.507-1.188) | 0.243 |  |
| *P* for trend |  | 0.432 |  |  | 0.285 |  |  | 0.580 |  |
| **Ethnic minorities** | |  |  |  |  |  |  |  |  |
| <2000 m | 1 |  |  | 1 |  |  | 1 |  |  |
| 2000-2499 m | 2.499  (1.857-3.636) | <0.001 |  | 2.474  (1.828-3.347) | <0.001 |  | 2.167  (1.595-2.945) | <0.001 |  |
| 2500-2999 m | 2.170  (1.600-2.942) | <0.001 |  | 2.156  (1.583-2.935) | <0.001 |  | 1.682  (1.228-2.303) | 0.001 |  |
| ≥3000 m | 3.270  (2.237-4.781) | <0.001 |  | 3.189  (2.182-4.663) | <0.001 |  | 2.704  (1.828-3.998) | <0.001 |  |
| *P* for trend |  | <0.001 |  |  | <0.001 |  |  | <0.001 |  |
| **High SBP (≥140 mm Hg)** | |  |  |  |  |  |  |  | 0.008 |
| <2000 m | 1 |  |  | 1 |  |  | 1 |  |  |
| 2000-2499 m | 1.343 (1.156-1.559) | <0.001 |  | 1.349 (1.163-1.564) | <0.001 |  | 1.292  (1.115-1.497) | 0.001 |  |
| 2500-2999 m | 1.620 (1.295-2.028) | <0.001 |  | 1.607 (1.289-2.004) | <0.001 |  | 1.400  (1.114-1.761) | 0.004 |  |
| ≥3000 m | 1.741 (1.407-2.152) | <0.001 |  | 1.721 (1.390-2.130) | <0.001 |  | 1.609  (1.296-1.997) | <0.001 |  |
| *P* for trend | | <0.001 |  |  | <0.001 |  |  | <0.001 |  |
| **Low SBP (<140 mmHg)** | |  |  |  |  |  |  |  |  |
| <2000 m | 1 |  |  | 1 |  |  | 1 |  |  |
| 2000-2499 m | 1.121 (0.966-1.300) | 0.132 |  | 1.164 (1.005-1.347) | 0.043 |  | 1.154 (0.995-1.337) | 0.058 |  |
| 2500-2999 m | 0.943 (0.760-1.169) | 0.592 |  | 0.940 (0.761-1.161) | 0.567 |  | 0.766 (0.612-0.957) | 0.019 |  |
| ≥3000 m | 1.102 (0.886-1.372) | 0.384 |  | 1.088 (0.878-1.349) | 0.440 |  | 1.003 (0.806-1.247) | 0.981 |  |
| *P* for trend | | 0.069 |  |  | 0.068 |  |  | 0.089 |  |

Note: VHD, valvular heart disease; PR, prevalence ratio; SBP, systolic blood pressure.

# Table S6. Etiological distribution of 129 patients with clinically significant VHD

| **Etiology** | **Total** | **<2000 m** | **2000-2499 m** | **2500-2999 m** | **≥3000 m** |
| --- | --- | --- | --- | --- | --- |
| Functional | 54 (41.9) | 19 (44.2) | 6 (28.6) | 13 (28.2) | 16 (84.2) |
| Degenerative | 70 (54.2) | 21 (48.9) | 14 (66.7) | 32 (69.6) | 3 (15.8) |
| Congenital | 1 (0.8) | 1 (2.3) | 0 (0.0) | 0 (0.0) | 0 (0.0) |
| Rheumatic | 3 (2.3) | 1 (2.3) | 1 (4.7) | 1 (2.2) | 0 (0.0) |
| Others | 1 (0.8) | 1 (2.3) | 0 (0.0) | 0 (0.0) | 0 (0.0) |

Note: Data are presented as numbers and percentages (%), indicate the distribution of etiologies in different altitude groups.

# Table S7. Etiological distribution of patients with clinically significant MR and TR

| **Etiology** | **MR (n=32)** | **TR (n=34)** |
| --- | --- | --- |
| Functional | 27 (84.4) | 34 (100.0) |
| Degenerative | 4 (12.5) | 0 (0.0) |
| Congenital | 0 (0.0) | 0 (0.0) |
| Rheumatic | 1 (3.1) | 0 (0.0) |
| Others | 0 (0.0) | 0 (0.0) |

Note: Data are presented as numbers and percentages (%). MR, mitral regurgitation; TR, tricuspid regurgitation.

# Reference

1. Lang RM, Badano LP, Mor-Avi V, et al. Recommendations for cardiac chamber quantification by echocardiography in adults: an update from the American Society of Echocardiography and the European Association of Cardiovascular Imaging. J Am Soc Echocardiogr. 2015;28 (1):1-39.e14. doi:10.1016/j.echo.2014.10.003
2. Merlos P, Núñez J, Sanchis J, et al. Echocardiographic estimation of pulmonary arterial systolic pressure in acute heart failure. Prognostic implications. Eur J Intern Med 2013, 24 (6):562-567.
3. Melzig C, Wörz S, Egenlauf B, , et al. Combined automated 3D volumetry by pulmonary CT angiography and echocardiography for detection of pulmonary hypertension. Eur Radiol 2019, 29 (11):6059-6068.
4. Huang X, Dhruva SS, Yuan X, et al. Characteristics, interventions and outcomes of patients with valvular heart disease hospitalised in China: a cross-sectional study. BMJ Open 2021, 11 (11):e052946.
5. Xu H, Liu Q, Cao K, et al. Distribution, Characteristics, and Management of Older Patients With Valvular Heart Disease in China: China-DVD Study. JACC Asia 2022, 2 (3):354-365.
6. Ou Q, Chen Y, Yu S, Guo X, Zhao H, Sun Y. Prevalence of left atrial enlargement and its risk factors in general Chinese population. BMC Cardiovasc Disord. 2016;16:53.
7. Lang RM, Bierig M, Devereux RB, et al. Recommendations for chamber quantification: a report from the American Society of Echocardiography's Guidelines and Standards Committee and the Chamber Quantification Writing Group, developed in conjunction with the European Association of Echocardiography, a branch of the European Society of Cardiology. J Am Soc Echocardiogr. 2005;18(12):1440-1463. doi:10.1016/j.echo.2005.10.005.
8. Yaghi S, Moon YP, Mora-McLaughlin C, et al. Left atrial enlargement and stroke recurrence: the Northern Manhattan Stroke Study. Stroke. 2015;46(6):1488-1493. doi:10.1161/STROKEAHA.115.008711.
9. Zheng C, Wang X, Tang H, et al. Habitation Altitude and Left Ventricular Diastolic Function: A Population-Based Study. J Am Heart Assoc. 2021;10(3):e018079.
